# Femoral Varus Osteotomy for Hip Instability after Traumatic Fracture Dislocations of the Hip Associated with Femoral Head Fractures: A Report of Two Cases

**DOI:** 10.1155/2016/1450842

**Published:** 2016-05-16

**Authors:** Shuichi Miyamoto, Junichi Nakamura, Satoshi Iida, Chiho Suzuki, Seiji Ohtori, Sumihisa Orita, Kazuhisa Takahashi

**Affiliations:** ^1^Graduate School of Medicine, Chiba University, 1-8-1 Inohana, Chuo-ku, Chiba City, Chiba 260-8677, Japan; ^2^Matudo City Hospital, 4005 Kamihongou, Matudo City, Chiba 271-8511, Japan

## Abstract

Fracture of the femoral head and the acetabulum with traumatic dislocation of the hip is a severe injury representing various types and unfavorable outcome. We showed a 45-year-old man with Pipkin type-IV fracture and coxa valga. An immediate closed reduction was achieved followed by open reduction and internal fixation via a posterior approach 6 days later. However, dislocation occurred three times without traumatic events after three weeks. CT demonstrated no displacement of posterior fragments or implant failure. Femoral intertrochanteric varus osteotomy was performed to gain concentric stability and successfully resolved recurrent dislocation. Another 45-year-old woman with Pipkin type-IV fracture and coxa valga also underwent closed reduction initially and then continued conservative treatment. After eight weeks, when she started gait training, progressive pain became symptomatic. Persistent hip pain at weight bearing was not improved in spite of arthroscopic synovectomy and osteochondroplasty. Two years after injury, femoral intertrochanteric varus osteotomy was indicated and her refractory pain was resolved gradually. We suggest that femoral varus osteotomy should be considered for superolateral subluxation associated fracture dislocation of the hip in Pipkin type-IV and coxa valga.

## 1. Introduction

Fractures of the femoral head are the result of high-energy trauma, concomitant with traumatic dislocation of the hip and fractures of the acetabulum. The femoral head may be fractured with an incidence of 6%–15% of traumatic hip dislocations [1–3], when it faces the edge of the posteroinferior acetabular wall such as a dashboard injury. It was first described by Birkett in 1869 [4] and classified by Pipkin [5]; however, diagnosis and treatment of fracture dislocations of the hip remain controversial. Treatment options include conservative treatment, excision of fracture fragments, open reduction and internal fixation, arthroplasty, and arthrodesis. Early diagnosis and accurate reduction are necessary for the successful outcome of surgery for fracture dislocation of the hip [6]. However, there is no consensus on the surgical approach and fixation techniques [3, 7, 8]. Complications associated with this injury are avascular necrosis of the femoral head, nonunion of the femoral head fragment, and posttraumatic osteoarthritis [1, 5, 9, 10].

We report the cases of two patients with a Pipkin type-IV fracture (fracture of the femoral head cephalad to the fovea associated with fracture of the acetabular rim) and treatment for the hip instability.

## 2. Case Presentation

### 2.1. Case 1

**Figure 1:**
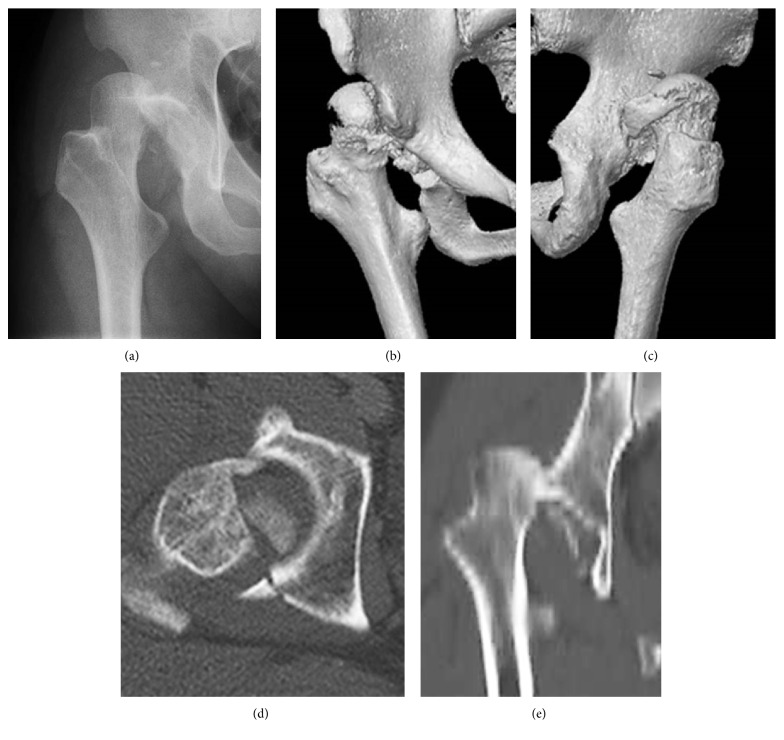
Case 1. A 45-year-old man with Pipkin type-IV fracture of the right hip with posterior dislocation of the femoral head. (a) Anteroposterior X-ray image of the right hip at the injury. (b) Anteroposterior three-dimensional computed tomography showing dislocation and fracture of the femoral head. (c) Posteroanterior three-dimensional computed tomography showing displacement of the posterosuperior wall and rim. (d) Axial computed tomography showing fracture of the femoral head and posterior wall of the acetabulum. (e) Coronal computed tomography showing dislocation and fracture of femoral head.

**Figure 2:**
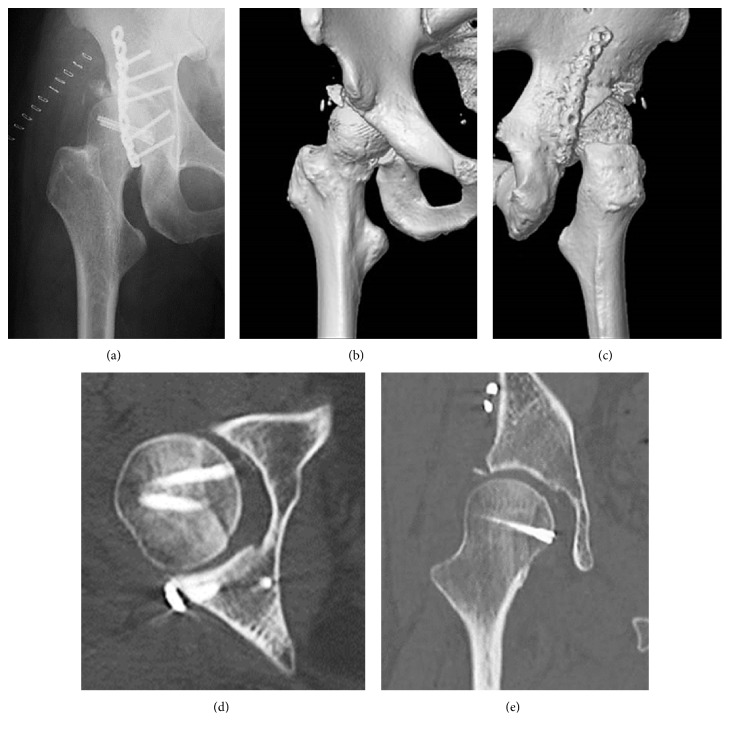
Case 1. (a) Anteroposterior X-ray image of the right hip after open reduction and internal fixation. (b) Anteroposterior three-dimensional computed tomography showing acceptable reduction of the femoral head. (c) Posteroanterior three-dimensional computed tomography showing acceptable reduction of the posterosuperior wall. (d) Axial computed tomography showing acceptable internal fixation of the femoral head and posterior wall. (e) Coronal computed tomography showing acceptable internal fixation of the femoral head and congruity.

A 45-year-old, previously healthy man was involved in a road traffic accident. X-ray imaging showed a Pipkin type-IV fracture of the right femoral head with posterior dislocation (Figures 1(a)–1(e)). He also had a traumatic cerebrovascular disorder. In the emergency room, an immediate closed reduction of the right hip was attempted with spinal anesthesia followed by direct traction of his distal femur. At 6 days after injury, he underwent open reduction and internal fixation (ORIF) via a posterior approach [11] in a left decubitus position. Surgical dislocation was applied using two 3.5-millimeter Herbert screws for the femoral head fracture and reconstruction plate and cortex screws for posterior fragments of the acetabulum. Postoperative X-ray imaging and computed tomography (CT) showed acceptable reduction of the femoral head and acetabular fragment (Figures 2(a)–2(e)). Passive range of motion in the hip joint was allowed at one week after ORIF and wheelchair with non-weight-bearing movement was permitted from 2 weeks. At 3 weeks, he moved to a rehabilitation hospital.

**Figure 3:**
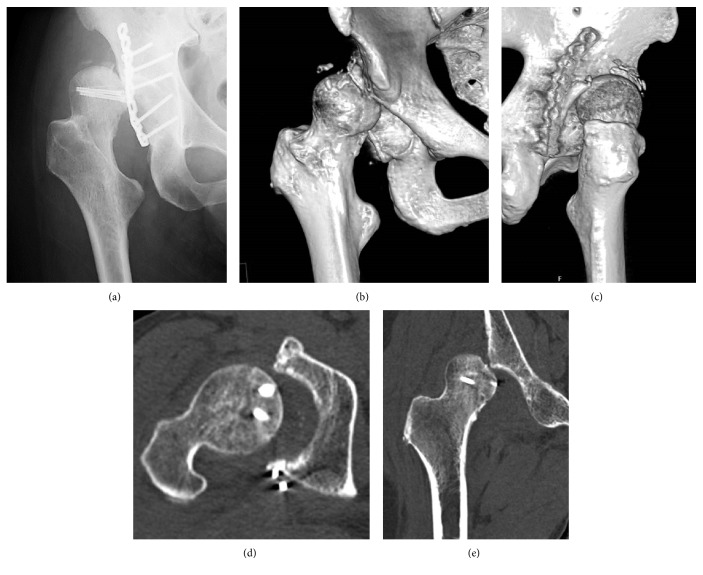
Case 1. (a) Anteroposterior X-ray image of the right hip at the superolateral dislocation. (b) Anteroposterior three-dimensional computed tomography showing superolateral dislocation of the femoral head and a bony defect of the superior part of the acetabular rim. (c) Posteroanterior three-dimensional computed tomography showing no displacement of the posterior fragments or implant failures. (d) Axial computed tomography showing irregular congruity, no displacement of the femoral head or posterior wall. (e) Coronal computed tomography showing superolateral dislocation of the femoral head and no displacement of the femoral head.

**Figure 4:**
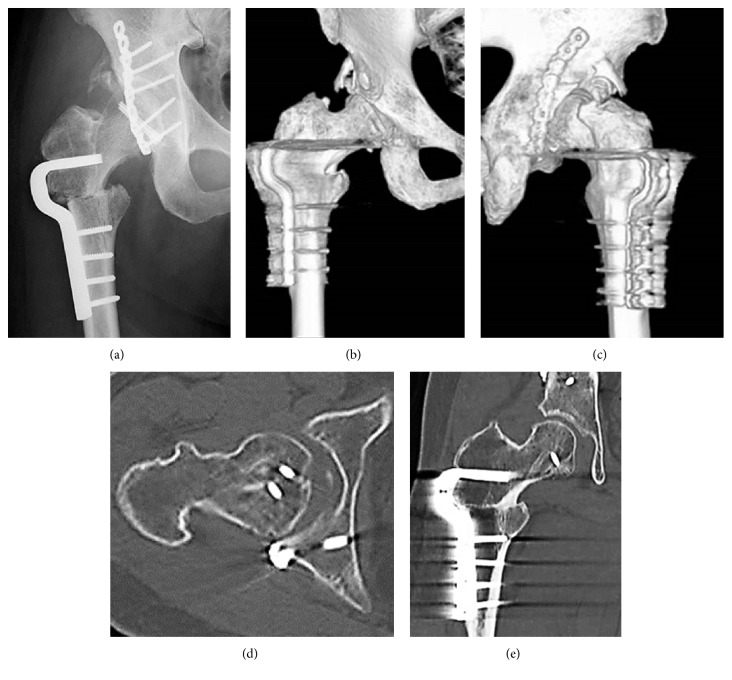
Case 1. (a) Anteroposterior X-ray image of the right hip after a Pauwel intertrochanteric varus osteotomy. (b) Anteroposterior and (c) posteroanterior three-dimensional computed tomography showing derotation of 35° and varus of 30°. (d) Axial image and (e) coronal computed tomography showing congruity was improved and internal fixation was stable.

**Figure 5:**
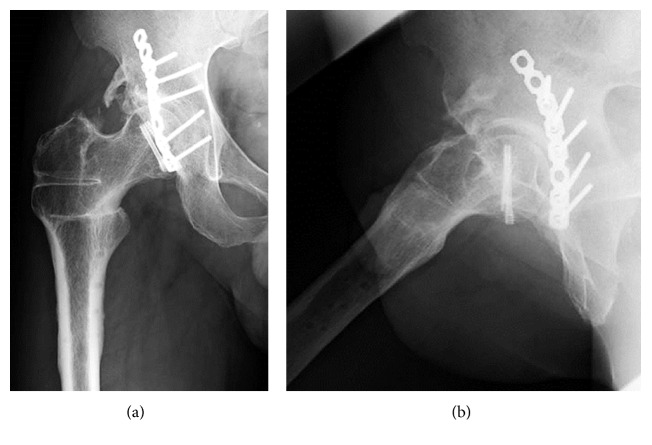
Case 1. (a) Anteroposterior X-ray image of the right hip at 3 years after osteotomy. (b) Lateral X-ray image of the right hip at 3 years after osteotomy.

However, he returned because of three superolateral dislocations without traumatic events. His right hip was easily dislocated with adduction and external rotation, while the reduced position was stabilized with 30° degrees of abduction and internal rotation. X-ray imaging showed coxa valga with a femoral neck angle of 160° and 30° of anteversion. CT demonstrated a bony defect of the superior part of the acetabular rim without displacement of posterior fragments or implant failures (Figures 3(a)–3(e)). At 6 weeks after ORIF, a Pauwels intertrochanteric varus osteotomy [12] was performed to stabilize the superolateral instability. The blade was accorded with the anteversion of the femoral neck. Derotation of 35° and varus of 30° were chosen using the intraoperative stability of the hip (Figures 4(a)–4(e)). As a result, the proximal femur was corrected to a femoral neck angle of 130° and 5° of retroversion. Operation time was 157 minutes, intraoperative blood loss was 880 mL, hospital charge was 14,000 dollars, and duration of hospital stays was 70 days. At three years after osteotomy, the hip has been stable without further dislocation, but with slight pain. Postoperative Harris hip score showed pain in 40 points, function in 26 points, activity in 4 points, absence of deformity in 4 points, range of motion in 4 points, and total in 78 points. A radiograph showed concentric reduction with bone healing (Figure 5).

### 2.2. Case 2

A 45-year-old woman was injured in a traffic accident and was transported to the emergency room. She was diagnosed as having a Pipkin type-IV fracture of her right hip with posterior dislocation of the femoral head with sciatic nerve palsy. Closed reduction was used to treat her dislocation as soon as possible and was retained with direct traction of the distal femur. She underwent conservative treatment because the displacement of the fragments was acceptable. After 8 weeks, she started gait training by partial-weight bearing on two crutches. However, she had progressive pain and complained of instability of her right hip when she walked.

**Figure 6:**
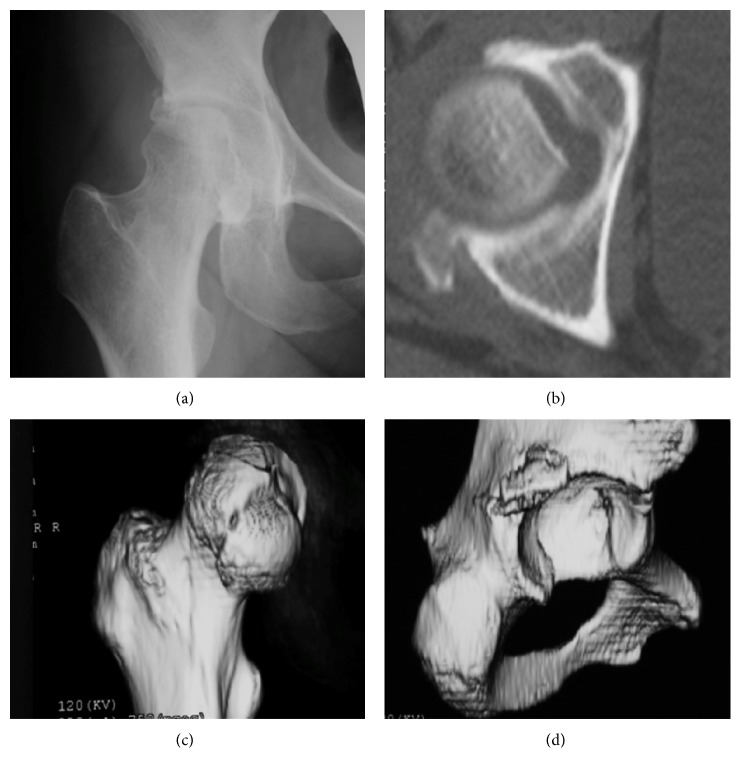
Case 2. A 45-year-old woman with Pipkin type-IV fracture of the right hip with posterior dislocation of the femoral head. (a) Anteroposterior X-ray image of the right hip after closed reduction, (b) axial computed tomography showing a defect of the femoral head and posterior wall of the acetabulum. (c) Three-dimensional image of the femur revealing displacement of a medial fragment of the femoral head. (d) Three-dimensional image of the acetabulum showing displacement of posterosuperior wall.

**Figure 7:**
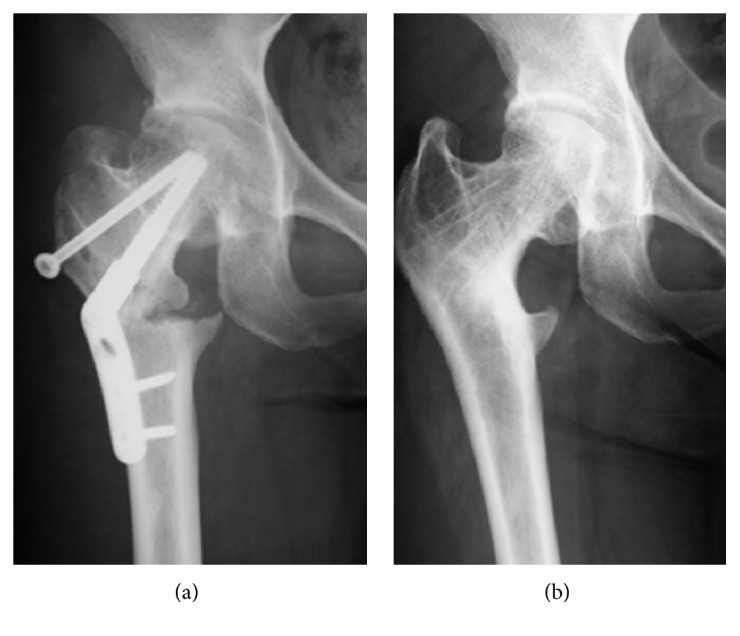
Case 2. (a) Anteroposterior X-ray image of the right hip after femoral curved varus osteotomy, (b) 9 years after osteotomy, X-ray image showing a spherical femoral head with joint space and remodeling of the femoral neck.

She was referred to our hospital at 3 months after her injury. She was treated with combinations of nonsteroidal anti-inflammatory drugs and physical therapy for range of motion and muscle strengthening, without improvement. Then, she underwent arthroscopic synovectomy at 5 months after injury. However, she still complained of persistent right hip pain. At 8 months after injury, osteochondroplasty was performed to excise the bony bump at the anteromedial part of the fragment of the femoral head (Figures 6(a)–6(d)) and to restore the original sphericity, using a Smith-Petersen approach in a supine position. Nevertheless, the right hip pain showed no improvement with crutches. She went to a pain clinic for epidural anesthesia. Two years after injury, femoral curved varus osteotomy [13] was indicated to restore her unstable hip with 25° of varus and 0° of anteversion/retroversion correction (Figure 7(a)). Operation time was 140 minutes, intraoperative blood loss was 486 mL, hospital charge was 34,000 dollars, and duration of hospital stays was 110 days. Her intractable hip pain was gradually resolved and she could walk without crutches at 6 years postoperatively. At 9 years after osteotomy, she had slight pain without the joint space narrowing (Figure 7(b)). Postoperative Harris hip score showed pain in 40 points, function in 23 points, activity in 4 points, absence of deformity in 4 points, range of motion in 4 points, and total in 75 points.

## 3. Discussion

Fracture type is predictive for the outcome of fracture dislocation of the hip [1–3]. For Pipkin classification, the femoral head fracture at the non-weight-bearing portion (types I and II) was favorable [5] and 31% of types III (with femoral neck fracture) and IV (with acetabular fracture) were poor [14]. By contrast, Nast-Kolb et al. recommended surgical treatment for all types [15]. Particularly, Pipkin type III fractures are associated with poor prognosis, so total hip arthroplasty is recommended [16]. In case 2, the initial treatment was closed reduction and the outcome was poor.

Anterolateral (Watson-Jones) [17], anterior (Smith-Petersen) [18], and posterior (Kocher-Langenbeck) [19] surgical approaches to this type of trauma have been proposed. However, which approach should be used remains controversial.

A posterior approach is generally recommended because it will allow direct visualization of the posterior wall of the acetabulum and protection of sciatic nerve and artery [1, 20, 21]. By contrast, Swiontkowski et al. [10] suggested that an anterior approach could reduce the operative time and blood loss and improve visualization. A transtrochanteric approach also provided visualization and protection of the blood supply of the femoral head [22, 23]. We used a posterior approach in case 1.

Whether a preserved bony fragment should be excised or internally fixed is a matter for debate. Small fragments of the femoral head measuring less than 1 cm could be removed, while larger fragments should be fixed in all types of femoral head fractures [9, 16, 24]. In less than about 30% of the femoral head type-I and type-II fractures, the fragment is too small to be fixed [21]. Arthroscopy is an option for extracting small intra-articular fragments [25, 26]. Lederer et al. argued that removal was better than osteosynthesis, because 20%–24% of patients had to undergo total hip arthroplasty [2, 27]. Similar results were obtained with both treatments [24, 28].

The safe interval between injury and reduction is controversial. Early reduction within 6, 12, and 24 hours provided a better result than delayed reduction [21, 28, 29]. Giannoudis et al. [3] reported that the incidence of major late complications included osteonecrosis (11.9%), posttraumatic arthritis (20%), and heterotopic ossification (16.8%) in their review of the literature. In both cases 1 and 2, initial reductions were achieved within 6 hours and there was no evidence of the complications described above.

Chronic recurrent dislocation of unstable hips is rare after fracture dislocations of the hip [30]. The case of a patient with a Pipkin type-I fracture treated with open reduction and excision of fragments has been reported. Four weeks after the injury, the patient had pain in the left hip with clicking and an inability to stand. Three months later, posterior dislocations occurred five times with flexion of the hip. This was eventually resolved by surgical treatment with a posterior acetabuloplasty and femoral varus osteotomy with a favorable outcome. CT demonstrated that hips with <34% of the remaining posterior acetabulum were unstable and with >55% were stable after posterior fracture dislocation of the hip [31].

In this report, two patients had chronic recurrent dislocation and persistent hip pain at weight bearing because of an unstable hip. Both patients had the same Pipkin type-IV fracture and coxa valga: femoral neck angle was 160° in case 1 and 159° in case 2. Both patients successfully gained stability with varus osteotomy, consistent with the findings of Marti and Kloen [30].

In conclusion, fracture dislocation of the hip in Pipkin type-IV and coxa valga are associated with unstable hips. Femoral varus osteotomy could stabilize the superolateral subluxation.

## References

[1] Thompson V. P.,  Epstein H. C. (1951). Traumatic dislocation of the hip; a survey of two hundred and four cases covering a period of twenty-one years. *The Journal of Bone and Joint Surgery*.

[2] Tonetti J., Ruatti S., Lafontan V. (2010). Is femoral head fracture-dislocation management improvable: a retrospective study in 110 cases. *Orthopaedics and Traumatology: Surgery and Research*.

[3] Giannoudis P. V., Kontakis G., Christoforakis Z., Akula M., Tosounidis T., Koutras C. (2009). Management, complications and clinical results of femoral head fractures. *Injury*.

[4] Birkett J. (1869). Description of a dislocation of the head of the femur, complicated with its fracture; with remarks. *Medico-Chirurgical Transactions*.

[5] Pipkin G. (1957). Treatment of grade IV fracture-dislocation of the hip. *The Journal of Bone & Joint Surgery—American Volume*.

[6] Şahin V., Karakaş E. S., Aksu S., Atlihan D., Turk C. Y., Halici M. (2003). Traumatic dislocation and fracture-dislocation of the hip: a long-term follow-up study. *Journal of Trauma-Injury Infection & Critical Care*.

[7] Chen Z.-W., Lin B., Zhai W.-L. (2011). Conservative versus surgical management of Pipkin type I fractures associated with posterior dislocation of the hip: a randomised controlled trial. *International Orthopaedics*.

[8] Prokop A., Helling H.-J., Hahn U., Udomkaewkanjana C., Rehm K. E. (2005). Biodegradable implants for pipkin fractures. *Clinical Orthopaedics and Related Research*.

[9] Butler J. E. (1981). Pipkin type-II fractures of the femoral head. *Journal of Bone and Joint Surgery—Series A*.

[10] Swiontkowski M. F., Thorpe M., Seiler J. G., Hansen S. T. (1992). Operative management of displaced femoral head fractures: case-matched comparison of anterior versus posterior approaches for pipkin i and pipkin ii fractures. *Journal of Orthopaedic Trauma*.

[11] Moore A. T. (1957). The self-locking metal hip prosthesis. *The Journal of Bone and Joint Surgery. American Volume*.

[12] Pauwels F. (1950). Treatment of coxa valga luxans. *Zeitschrift für Orthopädie und Ihre Grenzgebiete*.

[13] Sakano S., Hasegawa Y., Torii Y., Kawasaki M., Ishiguro N. (2004). Curved intertrochanteric varus osteotomy for osteonecrosis of the femoral head. *The Journal of Bone & Joint Surgery—British Volume*.

[14] Marchetti M. E., Steinberg G. G., Coumas J. M. (1996). Intermediate-term experience of Pipkin fracture-dislocations of the hip. *Journal of Orthopaedic Trauma*.

[15] Nast-Kolb D., Ruchholtz S., Schweiberer L. (1997). Treatment of Pipkin fractures. *Orthopade*.

[16] Kokubo Y., Uchida K., Takeno K. (2013). Dislocated intra-articular femoral head fracture associated with fracture-dislocation of the hip and acetabulum: report of 12 cases and technical notes on surgical intervention. *European Journal of Orthopaedic Surgery and Traumatology*.

[17] Stockenhuber N., Schweighofer F., Seibert F. J. (1994). Diagnosis, therapy and prognosis of Pipkin fractures (femur head dislocation fractures). *Chirurg*.

[18] Stannard J. P., Harris H. W., Volgas D. A., Alonso J. E., Rodríguez-Merchán E. C., Goddard N. J. (2000). Functional outcome of patients with femoral head fractures associated with hip dislocations. *Clinical Orthopaedics and Related Research*.

[19] Özcan M., Çopuroğlu C., Sarıdoğan K. (2011). Fractures of the femoral head: what are the reasons for poor outcome?. *Ulusal Travma ve Acil Cerrahi Dergisi*.

[20] Garland D. E., Miller G. (1984). Fractures and dislocations about the hip in head-injured adults. *Clinical Orthopaedics and Related Research*.

[21] Epstein H. C., Wiss D. A., Cozen L. (1985). Posterior fracture dislocation of the hip with fractures of the femoral head. *Clinical Orthopaedics and Related Research*.

[22] Mostafa M. F., El-Adl W., El-Sayed M. A.-E. (2014). Operative treatment of displaced Pipkin type I and II femoral head fractures. *Archives of Orthopaedic and Trauma Surgery*.

[23] Chen Z., Dai Z., Liao Y., Fan W., Tang Z. (2011). Treatment of type IV Pipkin fracture through transtrochanteric approach with trochanteric osteotomy. *Zhongguo Xiu Fu Chong Jian Wai Ke Za Zhi*.

[24] Droll K. P., Broekhuyse H., O'Brien P. (2007). Fracture of the femoral head. *The Journal of the American Academy of Orthopaedic Surgeons*.

[25] Yamamoto Y., Ide T., Ono T., Hamada Y. (2003). Usefulness of arthroscopic surgery in hip trauma cases. *Arthroscopy*.

[26] Mullis B. H., Dahners L. E. (2006). Hip arthroscopy to remove loose bodies after traumatic dislocation. *Journal of Orthopaedic Trauma*.

[27] Lederer S., Tauber M., Karpik S., Bogner R., Auffarth A., Resch H. (2007). Fractures of the femoral head. A multicenter study. *Unfallchirurg*.

[28] Chen Z.-W., Zhai W.-L., Ding Z.-Q. (2011). Operative versus nonoperative management of Pipkin type-II fractures associated with posterior hip dislocation. *Orthopedics*.

[29] McMurtry I. A., Quaile A. (2001). Closed reduction of the traumatically dislocated hip: a new technique. *Injury*.

[30] Marti R. K., Kloen P. (2000). Chronic recurrent posterior dislocation of the hip after a Pipkin fracture treated with intertrochanteric osteotomy and acetabuloplasty. A case report. *The Journal of Bone & Joint Surgery—American Volume*.

[31] Calkins M. S., Zych G., Latta L., Borja F. J., Mnaymneh W. (1988). Computed tomography evaluation of stability in posterior fracture dislocation of the hip. *Clinical Orthopaedics and Related Research*.

